# Congenital heart disease diagnosis using machine learning: a systematic literature review

**DOI:** 10.3389/fmed.2026.1757468

**Published:** 2026-04-21

**Authors:** Khalil Khan, Ikram Syed, Farhan Ullah, Rehan Ullah Khan

**Affiliations:** 1 Department of Information Technology, College of Computer, Qassim University, Buraydah, Saudi Arabia; 2 Department of Information and Communication Engineering, Hankuk University of Foreign Studies, Seoul, Republic of Korea; 3 School of Internet of Things Engineering, Wuxi University, Wuxi, China

**Keywords:** artificial intelligence, congenital heart disease, deep learning, health care, machine learning

## Abstract

Congenital heart disease is among the most common fetal abnormalities and birth defects. Despite identifying numerous risk factors influencing its onset, a comprehensive understanding of its genesis and management across diverse populations remains limited. Recent advancements in machine learning have demonstrated the potential for leveraging patient data to enable early congenital heart disease detection. Over the past seven years, researchers have proposed various data-driven and algorithmic solutions to address this challenge. This paper presents a systematic review of congenital heart disease recognition using machine learning, conducting a meta-analysis of 432 references from leading journals published between 2018 and 2025. A detailed investigation of 74 scholarly works highlights key factors, including databases, algorithms, applications, and solutions. Additionally, the survey outlines reported datasets used by machine learning experts for congenital heart disease recognition. Using a systematic literature review methodology, this study identifies critical challenges and opportunities in applying machine learning to congenital heart disease.

## 1 Introduction

Congetial heart disease (CHD) is a major birth defect with the highest mortality rate worldwide, with a reported incidence of approximately 1.8 per 100 live births (1, 2). This disease can be properly cured if early detection and intervention are performed on time (3). Moreover, a timely and accurate diagnosis is crucial for affected children to receive effective treatment, significantly increasing the likelihood of successful surgical outcomes. Medical practitioners believe that early detection and surgical intervention of CHD in a timely manner lead to an asymptomatic CHD condition, which is a healthy condition (2, 4). However, due to a complex diagnosis process and the unavailability of pediatric cardiologists in remote areas, a significant number of critical CHDs continue to go undiagnosed (5).

CHD encompasses cardiac abnormalities and structural alterations of the heart that are present at birth. Each person with CHD has a unique heart, made up of a unique mix of heart problems that were there before surgery and changes that happened due to long-term remodeling (6). Researchers reported thirty-five different types of CHDs (7). Nanotologists and medical practitioners have not fully explored all these types. However, most doctors can identify easily five common types: Atrial septal defect (ASD), Ventricular Septal Defect (VSD), Patent ductus arteriosus (PDA), Patent Foramen Ovale (PFO), and Tetralogy of Fallot (TOF) (8). Doctors use various modalities such as Echocardiography (echo), Electrocardiography (ECG), Ultra sound (US), CT scan, X-rays, and Pulse Oximeter (POX) to diagnose CHDs. To the best of our understanding, the gold standard for CHD recognition is the echo.

The adoption of AI in medical diagnostics is rapidly expanding due to its efficiency, accessibility, and operational improvements (9–12). This growing integration has heightened the demand for AI in clinical settings. Recent developments in AI methodologies have inspired numerous studies on CHD, focusing on accelerating the scanning process, enhancing image quality, and improving diagnostic accuracy (13–15). By analyzing large datasets, AI can identify hidden patterns and relationships that might be overlooked by human experts. These insights enable AI models to deliver accurate predictions about the prevalence and severity of CHD. Researchers have also reported software applications that leverage AI for evaluating some ventricular functions through echo modality (16, 17). Despite these advancements, we are still distant from achieving a stage where AI can entirely substitute or autonomously assist medical professionals in the identification of CHD. AI can serve as a powerful tool to complement clinicians, but achieving reliable, autonomous AI-driven diagnosis requires overcoming significant technical and ethical challenges.

### 1.1 Research questions and key contributions

We conducted a systematic litrature review (SLR) with 422 research publications sourced from the Scopus database. We initially analyzed these publications' metadata, selecting 74 for an in-depth review. The metadata analysis aimed to address the following research questions:

- **RQ 1:** What are the global trends in CHD research contributions, including leading countries, institutions, and focus areas?
- **RQ 2:** How much do funding initiatives support research integration?

Subsequently, the detailed review of 74 papers focused on the following questions:

- **RQ 3:** What diagnostic methods do medical practitioners use to identify CHDs?
- **RQ 4:** What are the datasets reported in the literature for CHD recognition?
- **RQ 5:** What are the most commonly used machine learning (ML) algorithms for CHD recognition?
- **RQ 6:** What are the used evaluation measures, and what are the reported results on these standard datasets?

CHD-ML remains a relatively unexplored research area with limited comprehensive reviews available in the literature. Table 1 summarizes some previously published review articles on CHD-ML. While most papers reviewed ML algorithms (18–22), 80% lacked a discussion on evaluation metrics—an essential component for assessing model performance. Furthermore, only 30% of the reviewed papers included meta-analyses, highlighting a gap in systematically synthesizing findings. Notably, none of the reviewed articles provided a thorough overview of CHD datasets, which would be invaluable for computer vision (CV) or ML experts. Additionally, many of these reviews employed traditional literature review methods, lacking adherence to SLR methodologies (22, 23). Most research works also failed to specify the time-frames of the examined literature, as observed in Liu et al. (18), Pozza et al. (20), Jone et al. (24), Hoodbhoy et al. (25), Day et al. (22), and Oudkerk et al. (23). Our proposed study overcomes these limitations by adopting an SLR approach, offering a comprehensive analysis of CHD-ML encompassing datasets, algorithms, and evaluation metrics. The contributions of the proposed SLR are as follows:

- Our proposed article examines all aspects of CHD-ML by referencing over 74 papers published between 2018–2025. We systematically review and categorize recent advancements in each domain, presenting them in a coherent and accessible manner for readers.
- The SLR stands out as one of the most comprehensive analyses of CHD-ML, incorporating both a primary review and a targeted phase-2 review. To the best of our knowledge, it is the first SLR on CHD-ML to focus on specific research application domains and methodologies.
- The review provides a detailed summary of the modalities used by medical practitioners and cardiovascular experts for CHD recognition. Unlike previous reviews, this paper offers a nuanced analysis of these modalities and their practical applications.
- This study compiles and organizes all publicly available datasets relevant to CHD recognition, making it the first review to present a comprehensive inventory of datasets in this research area.
- The paper conducts a thorough analysis of ML methods applied to CHD recognition, highlighting their strengths and limitations. It spans a broad spectrum of approaches, from traditional ML techniques to the latest advancements in deep learning (DL) algorithms.
- Lastly, the study identifies and discusses emerging research directions in CHD-ML, providing valuable insights for both ML/DL experts and medical practitioners interested in advancing this critical field.

**Table 1:** Survey papers reported so far on CHD-ML.

S. No.	References	Time-period	ML-algorithms	Meta analysis	SLR
1	Liu et al. (18)	Not specified	✓	✕	✕
2	Ejaz et al. (19)	2015-2023	✓	✕	✕
3	Pozza et al. (20)	Not specified	✓	✕	✕
5	Sethi et al. (21)	2002–2022	✕	✓	✕
6	Jone et al. (24)	Not specified	✕	✓	✕
7	Hoodbhoy et al. (25)	Not specified	✕	✓	✓
8	Helman et al. (96)	2015-2018	✓	✕	✓
9	Day et al. (22)	Not specified	✓	✕	✕
10	Oudkerk et al. (23)	Not specified	✓	✕	✕

We argue that CHD-ML remains an underexplored area that requires significant attention from the research community. This survey focuses on papers published within the last seven years, offering a comprehensive analysis of the key factors necessary for developing cutting-edge CHD-ML systems. The SLR provides valuable insights into the end-to-end CHD-ML framework, enabling researchers to gain a holistic understanding of the field and facilitating structured exploration and development.

### 1.2 Organization of SLR

The primary objective of this SLR is to serve as a comprehensive reference for researchers and practitioners. It aims to provide an in-depth analysis of current trends and methods while identifying research gaps to facilitate the development of advanced ML and DL-based approaches for CHD recognition. The structure of this paper is illustrated in organized as follows: Section 2 outlines the SLR methodology, observations, and key findings are included in Section 3. Section 4 details the various diagnostic methods and databases employed for CHD recognition. Section 5 examines ML and DL algorithms, providing insights into their applications for CHD recognition. Section 6 presents a comprehensive discussion and future directions. The paper conclusion is presented in Section 7. This structure ensures a logical flow, guiding readers through the key components of the SLR and its findings.

## 2 Methods

An SLR involves the formulation of questions and the application of systematic and clear methodologies to identify, select, and critically evaluate relevant research papers from the studies reported so far (26). We select this method due to its accuracy and reliability in synthesizing academic material, as well as its widespread acceptance across several research disciplines. We perform the SLR in accordance with the PRISMA criteria. Although PRISMA is not a quality evaluation approach, it is extensively recognized in the research community for its evidence-based checklist items and four-phase analysis (27).

### 2.1 Data identification

**Figure 1:**
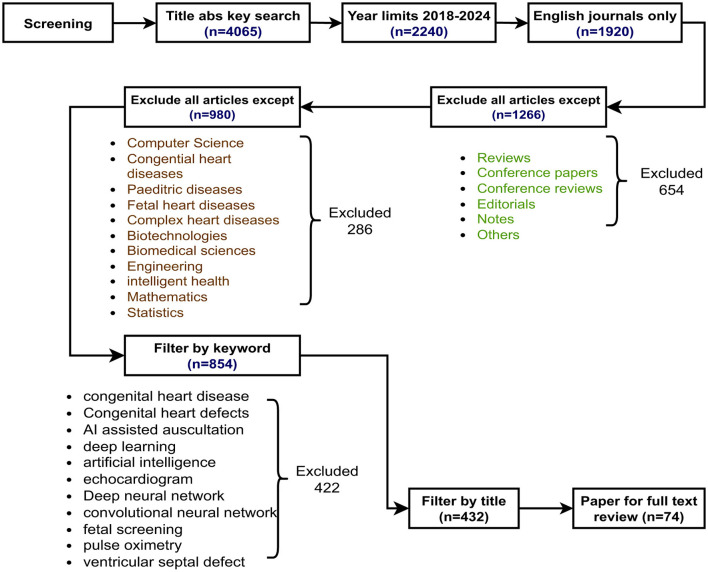
PRISMA flow diagram used in this research.

We conducted thorough research utilizing the Scopus integrated database, which encompasses all principal publishers, including Emerald, Elsevier, Springer, Taylor & Francis, IEEE, MDPI, Nature, and Wiley. The Scopus database is regarded as a reputable resource by numerous scholars for conducting SLR, owing to its high-quality indexed content (28). The search encompasses the period from January 2018 to October 2025 and incorporates all pertinent articles released throughout this timeframe. In our search for pertinent articles, we employed keywords such as “congential heart,” “machine learning,” “artificial intelligence,” and “congenital heart disease recognition.” Boolean operators are employed in conjunction with various keywords to expand the search area. The search procedure was devised. The survey includes each paper that combines empirical research with experimental results. We meticulously adhere to controlled vocabulary and associated keywords to effectively refine the search parameters. The comprehensive search approach is illustrated in Figure 1.

### 2.2 Initial data screening

The preliminary search in the Scopus database, utilizing specific keywords, yielded 4,065 articles. Following the implementation of the year limit from 2018 to 2025, the article count dropped to 2,240. The constraints of document type, language, subject area, and keywords reduce the total number of articles to 432. We identified 432 publications for screening based on their titles and abstracts after applying keywords and the search methodology. We imported data from 432 articles in Excel CSV format for further analysis. Duplicates were detected and eliminated utilizing Excel's duplicate functionality. We further reviewed the remaining 432 unique article titles and abstracts to ensure their inclusion. We evaluated 432 article titles and abstracts utilizing a standardized extraction form. We rejected studies that were unrelated to ML but pertinent to heart disease, or vice versa. After screening the titles, we thoroughly reviewed 74 articles, all of which satisfied the inclusion criteria. Figure 1 delineates the inclusion and exclusion criteria employed in this investigation.

## 3 Findings and observations

The results of the metadata analysis are presented in the next subsection. The results were obtained from a detailed analysis of 74 papers and then a metadata assessment of 432 journal articles. The metadata study included 432 papers classified by year, journals, authors, subject areas, funding sources, and institutions.

**Figure 2:**
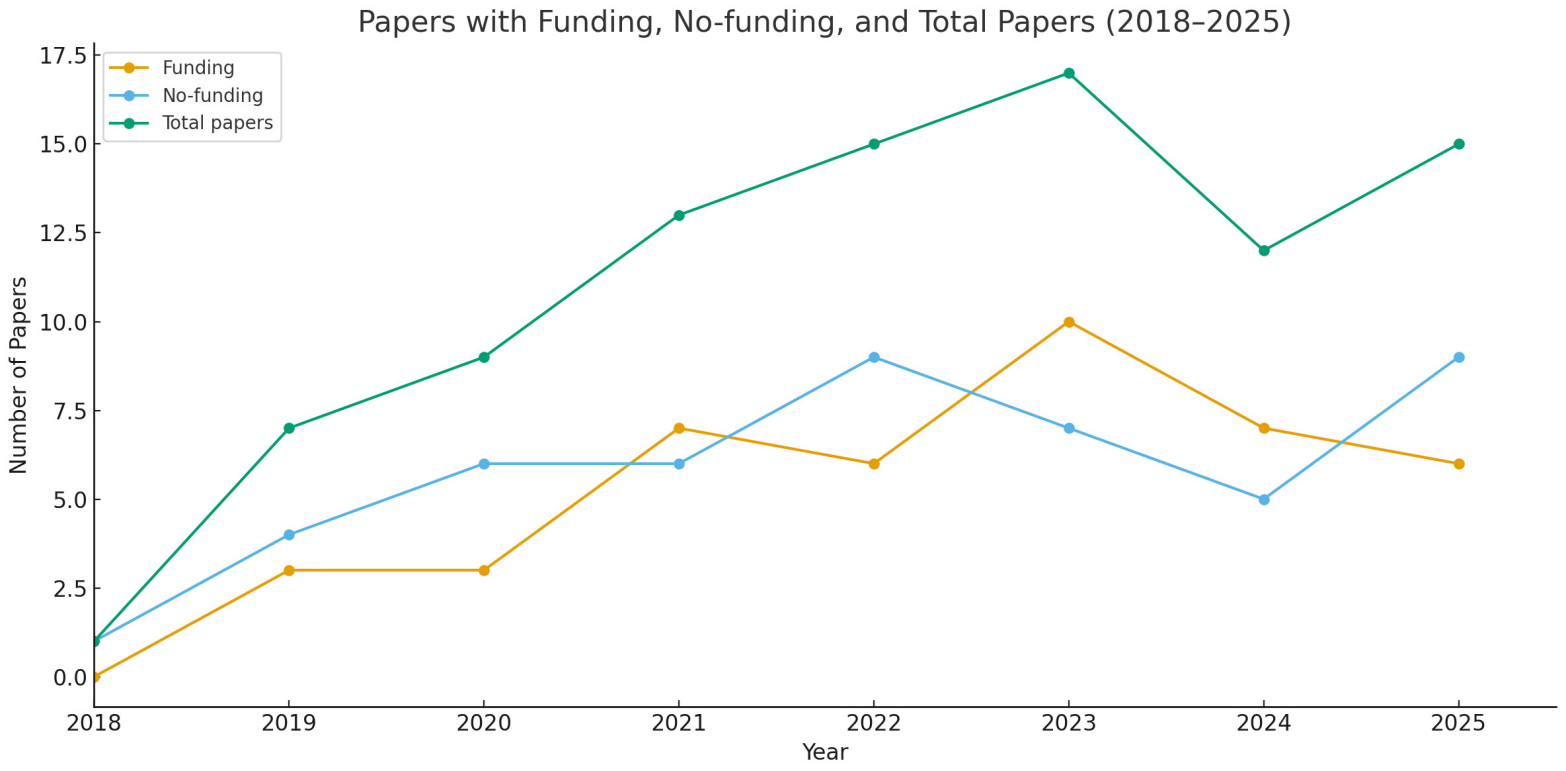
Total research papers published per year (2018–2025), papers with funding, and without funding.

The annual analysis of research articles on CHD recognition from 2018 to 2025 indicates a consistent upward trend, underscoring an increasing interest in the domain. Please see Figure 2 for details. The total number of papers exhibits a steady rise from 2018 (1 paper) to 2023 (17 papers), thereafter seeing a minor decrease in 2024 (12 papers). We can expect some more papers in 2025 since the year is not finished and there might be papers which are under review. Publications supported by funding shown a general increase over time, peaking at 10 publications in 2023. No-funding papers experienced initial growth from 2018 to 2022 but have since stabilized at approximately 6–9 papers annually in recent years. The rising involvement of funding indicates enhanced institutional and organizational backing for CHD research utilizing ML/DL, along with the wider acknowledgment of its significance.

Table 2 summarizes top 10 journals with work on CHD-ML. We do not observe frequent citations of CHD-ML in the literature. Frequent citation of research works provides a useful indicator of prominent contributors and impactful studies. It is worth noting that citation counts may vary slightly from Google Scholar due to differences in indexing methodologies and timelines. According to the data, the publication by Liang et al., focusing on the accurate diagnosis of CHD using AI, garnered the highest citation count with 620 citations. This is followed by Acharya et al., whose work on deep CNNs for diagnosing CHD using ECG signals received 326 citations. Other notable contributions include Arnaout et al. (206 citations) and Oliveira et al. (151 citations).

**Table 2:** Top 10 journals with work on CHD-ML.

S. No.	Journal	Impact factor
1	Nature medicine	58.7
2	European Heart Journal r	38.1
3	European Society of Cardiology	35.4
4	Journal of the American College of Cardiology	21.7
5	The Lancet Child & Adolescent Health	19.9
6	Nature Communication	14.7
7	Medical Image Analysis	10.6
8	Scientific data	9.8
9	IEEE Transactions of Medical Imaging	8.9
10	Future Generation Computer Systems	8.6

An analysis of these citation trends reveals two important insights. First, the number of citations across these papers is relatively low compared to more established fields, reflecting the nascent stage of CHD-ML as a research domain. Despite the emerging nature of this field, the variety of topics and methodologies in these studies underscores its interdisciplinary potential. Second, the data highlights a pressing need for further exploration, innovation, and dissemination of impactful findings in this domain. Fostering interdisciplinary collaborations between medical and technical researchers will be crucial in driving growth, improving adoption, and positioning CHD-ML as a mature and well-recognized research area.

**Figure 3:**
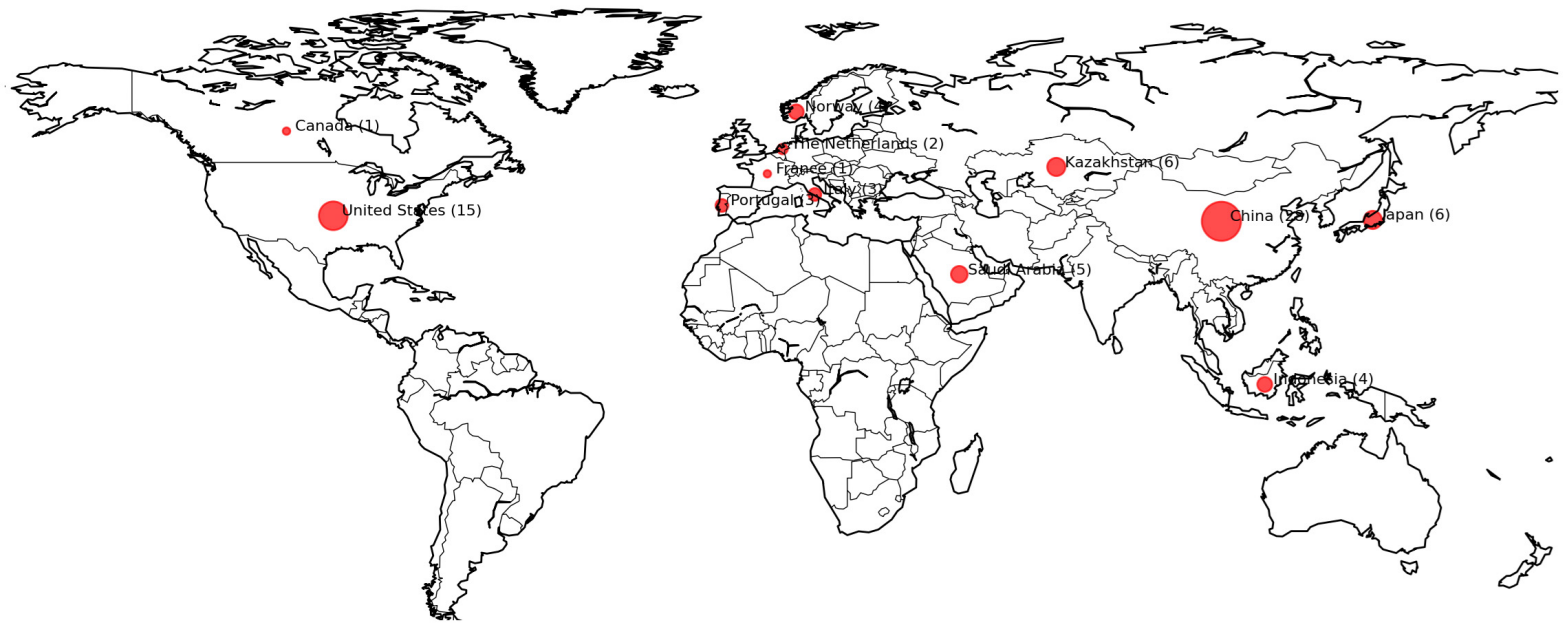
Countries that contributed in the form of publications to CHD-ML.

Figure 3 illustrates the contributions of research articles from various countries in CHD-ML. China accounts for the highest number of publications, contributing 46.3% of the total referenced works. The United States follows as the second-largest contributor, with 24.1%, highlighting its significant role in advancing this domain. Japan and Kazakhstan are tied for the third position, each contributing 5.6% of the total output. Other notable contributors include Saudi Arabia, Indonesia, Norway, Italy, and Portugal, each accounting for 3.7%, while smaller contributions come from the Netherlands, France, and Canada, each with 1.9%. These figures demonstrate China's dominance in CHD-ML research and reflect the USA's strong but secondary role in terms of the total publication share. The contributions from Japan, Kazakhstan, and other countries underscore the global interest in advancing this interdisciplinary field.

## 4 Datasets

The effectiveness of any ML-based approach heavily depends on the dataset used for experimental validation and evaluation. This section discusses the datasets developed to date for CHD recognition. Different diagnostic modalities are used by the medical practitioners to diagnose CHDs. The preliminary assessment for CHD in babies involves a combination of cardiac auscultation, POX, chest radiography, echo, and ECG. Diagnosing methods like X-rays, MRI of the heart, dual-source tomography scans, and CT examinations are very common; however, they are a lengthy complex process, expensive, and require qualified cardiologists (5). It is a fact that there is a common tendency of missing diagnosis relatively late, even when the condition warrants prompt management. In the following subsections, we discuss various modalities used for CHD recognition and their corresponding datasets reported in the literature.

### 4.1 Heart murmurs and PCG datasets

A heart murmur is an audible sound or vibration caused by turbulent blood flow within the heart. This murmurs can occur due to a variety of reasons, such as valve abnormalities, CHDs, or increased blood flow conditions (e.g., during pregnancy or fever). Murmurs are classified as either: Innocent (no underlying heart problem) and Abnormal (associated with structural heart issues). PCG is a tool used to record and analyze the sounds of the heart, including murmurs. A PCG device captures the heart's acoustic signals (such as normal heart sounds, S1 and S2 and ABNOR sounds such as gallops) and visualizes them in a graphical waveform. In summary, a heart murmur is a phenomenon or symptom, while PCG is a technique used to detect and analyze heart sounds, including murmurs.

AI has the potential to enhance the accuracy of auscultatory observations in the diagnosis of CHD. Heart disease recognition with ML in case of adults is somehow mature research area. A review article regarding this can be explored in Ismail et al. (29). Some research papers which use murmurs or PCG for CHD recognition can be explored in Thompson et al. (30), Xu et al. (31), Liu et al. (32), Yang et al. (33), Lv et al. (34), Belinha et al. (35), Ou et al. (36), and Jia (37). We must add here that the auscultatory findings of CHD are essential for clinical diagnosis and offer a cost-effective tool; however, they rely on clinical expertise, which poses a limitation in resource-constrained countries, necessitating objective and reportable support for clinicians, including peripheral health workers. The deficiency of skilled cardiologists at the peripheral level results in an inevitable delay in the clinical diagnosis of CHD, leading to postponed intervention and consequently a poor prognosis (38, 39). Advancements in AI for the detection of heart murmurs demonstrate potential sensitivity; nevertheless, clinical validation is necessary prior to broad clinical endorsement (40). Summary of the datasets for heart murmurs and PCG data is presented in Table 3.

**Table 3:** Datasets reported in literature for CHD.

Dataset	Year	CHD types	Access
PCG			
ZCHSound (32)	2022	NOR, ASD, VSD, PDA, PFA	✓
TCDD (41)	2015	—	✗
HSS (97)	2019	NOR, mild, severe	✗
Digiscope (98)	2017	—	✓
PhysioNet (83)	2016	NOR, ABNOR	✓
X-rays			
DICOM (99)	2022	NOR, VSD, ASD, PDA	✓
X-rays (43)	2022	NOR, ABNOR	✓
Echo			
Doppler (100)	2022	NOR, ASD, VSD	✓
CHDEcho (45)	2023	NOR, ASD, VSD	✓
CHD-Echo (44)	2021	NOR, ASD, VSD	✗
Seven-CHDs (101)	2022	Seven CHDs	✓
MRI			
HVSMR2-0	2024	NOR, ASD, VSD, PDA	✓
Complex-CHD (102)	2020	Complex CHDs	✓
Synthetic-CHDs (53)	2020	NOR, ASD, VSD, TOF	✓
ECG			
CHDdECG (57)	2023	NOR, ASD, VSD, PDA, TOF	✗
PhysioNet (83)	2024	NOR, ABNOR	✓
Fetuses-CHD (73)	2019	NOR, ASD, VSD	✗
POX			
POX (47)	2022	NOR, ABNOR	✗
Non-clinical			
Shanxi (59)	2020	NOR, ABNOR	✗
UCI (91)	2022	ASD, VSD, NOR	✓
Guangzhou (91)	2019	ABNOR, NOR	✓
CT images			
ImageCHD (103)	—	16 CHD types	✗

The ZCHSound (32) was collected at the Children's Hospital of Zhejiang University (ZU) and its affiliated facilities across China. ZU Kids Hospital is a prestigious institution dedicated to providing comprehensive pediatric healthcare services. Participation in the study was entirely voluntary, with explicit consent obtained from the participants' parents.

The TCD dataset (41) comprises heart sound recordings collected in 2014 (2014–2015). The total recordings are 5,282 obtained from 1,568 individuals, with an almost equal representation of males (787) and females (781). The participants ranged in age from infancy to young adulthood. These recordings, captured using a Littmann stethoscope, span 30 seconds each and underwent a quality assessment focused on murmur detection. For annotation accuracy, the data were reviewed by two cardiac specialist.

### 4.2 X-Rays

Despite advancements in imaging modalities, radiography still remains one of the most commonly utilized diagnostic imaging tests for patients with CHDs. X-rays are frequently employed to detect structural abnormalities in the hearts of infants. While X-rays provide valuable initial information, they are not sufficient for a full diagnosis.

The DICOM (42) consists of 828 radiograph files sourced from children, categorized into four groups: ASD, VSD, PDA, and NORM. Each chest radiograph associated with a particular cardiac issue is supported with a cardiac US report. Cardiologists gathered data on pediatric patients with CHDs who experienced hospitalisations. The interval between the cardiac US report and the chest radiograph does not exceed more than 3 days. Three certified cardiologists assessed the US findings and validated the diagnosis of CHD following the first storage of chest radiographs in DICOM format.

The Chest X-rays dataset proposed (43) consists of 3,255 frontal preoperative chest radiographs gathered retrospectively from patients under 18 years old between January 2018 and February 2022. The dataset comprised 1,174 radiographs of CHD cases and 2,081 non-CHD controls, with 142 CHD cases further classified as having pulmonary arterial hypertension associated with CHD. Photographs underwent a meticulous three-tier inspection procedure to guarantee quality, eliminating illegible, duplicate, or misidentified photographs. The preprocessing processes encompassed cropping, resizing to 448 × 448 pixels, random rotation, and normalization, with data augmentation implemented to mitigate class imbalance, especially for PAH-CHD cases.

### 4.3 Echocardiography

Echo and US are invaluable and most frequently used tools in diagnosing of CHD. Both rely on sound waves that further create images of heart internal structures, but echo is specifically tailored for the heart, offering detailed visualization of its chambers, valves, and blood flow. Through techniques like Doppler echo, clinicians can assess the direction and velocity of blood flow, helping to identify abnormalities such as septal defects, valve malformations, or improper circulation often associated with CHD.

Multiview CHD-echo (44) comprises a total of 1,308 children. Each patient has echo video recordings varying from 1 to 5 views. Furthermore, the physician chooses one high-quality 2D frame from each viewpoint in a film to compile a 2D echo dataset. During data acquisition, the patient was positioned supine, and the chest was exposed for the echo. The transducer frequency varied between 3 and 8 MHz. Utilizing the heart segmental technique, the locations of the heart, atria, and ventricles were determined, and the interrelations among the atria, ventricle, and aorta were examined. The atrial and ventricular septa were evaluated for anomalies, and the pulmonary venous return was analyzed. Five standard two-dimensional perspectives were obtained by the authors. All patients' diagnosis were corroborated by at least two prominent ultrasonography specialists.

CHDNets (45) consists of 5,840 echo recordings including three prevalent CHDs. Each echo film is associated with an individual patient and a specific visit. Keyframes in each echo film were meticulously selected by proficient specialist. Each echo was assessed using a three-tier evaluation approach. A first-level assessment was performed by a medical student possessing a bachelor's degree or above. Second-level evaluation was conducted by two junior echocardiographers, while third-level evaluation was carried out by two seasoned echocardiographers with over 10 years of clinical expertise. This tri-level evaluation approach guaranteed that each echo was accurately labeled with the appropriate diagnosis and cardiac defect localization. Upon finalizing the data labeling, 100 objects from the obtained dataset were chosen and reviewed by a third-party echocardiographist possessing over 20 years of clinical expertise. This strategy mitigate the influence and chances of human error the modeling process.

### 4.4 Pulse oximetery

POX is a technique that measures the oxygen saturation level in the blood. CHD screening with POX is being progressively implemented by medical practitioners in clinics since its inception (46). This strategy is also economical according to widely recognized standards (47, 48). This approach is not especially successful on an individual basis. It is used when combined with an additional diagnostic procedure. compared to other complex methods for diagnosis, it is extremely easy to use and yields results in a surprisingly quick time frame. POX screening can be performed easily within 24 h for each neonate. Typically, POX screening can overlook certain types of CHDs, and significant changes in oxygen saturation can go undetected in some instances. In contrast to echo, POX offers the most efficient strategy, taking only 2–3 min to verify the findings (49). single article using POX and ML for CHD recognition has been written by Huang et al. (50).

The POX dataset (50) was gathered throughout a two-year research period. The hospital documented a total of 44,147 births. The majority of infants were delivered at full term. A total of 498 newborns with CHD were initially found; 27 were identified using pulse oximetry screening, while 471 were detected with cardiac auscultation. This led to a total screening rate of 1.13% among the 44,147 live births. Out of the total cases, 458 neonates were assessed using ECG. This comprises 253 male newborns and 245 female infants. The principal types of CHDs included PDA (34.3%), ASD (20.5%), VSD (8.3%), and mixed anomalies of 34.5%.

The literature has reported HVSMR-2.0 as the latest dataset for CHD recognition. Researchers from BCH and MIT collaborated to compile the HVSMR-2.0 (51). The initial version of the same database was made available to the research community in 2016 (52). The HVSMR-2.0 comprises 3D CMR images obtained during standard clinical procedures. This dataset serves as a significant resource for investigating CHD, including instances with various cardiac anomalies, particularly those that have received treatments.

The dataset proposed in Diller et al. (53) consists of 6,400 long-axis MRI frames. All the patients had CHDs, particularly those with post-repair ToF, a common condition that causes the heart to turn blue. Fourteen centers in Germany participated in a state study that included MRIs. Patients over 8 years old who did not have implantable cardioverter-defibrillators met the inclusion criteria. The MRIs were saved in DICOM format and had information like the weight, height, and age. Data were made anonymous and were allowed to be used in a study in an ethical way.

### 4.5 Electrocardiogram

We believe that after echo, ECG is the second authentic signal for CHD diagnosis. Similar to echo, ECG diagnosis necessitates expert knowledge. The ECG identifies irregularities in cardiac rhythm and evaluates the occurrence of prior myocardial infarctions, ischemia, and other conditions that may precipitate heart failure. Due to its inherent safety and cost-effectiveness, ECG is a widely adapted tool for CHD diagnosis. Adult cardiology is very thoroughly explored by ML experts using ECG modality (54–56). In the following paragraphs, we discuss different datasets reported in the literature for CHD recognition using ECG.

An excellent dataset using ECG is proposed by Chen et al. (57). The 93,127 pediatric ECG cases in the dataset came from two hospitals in China: Guangdong Provincial People's Hospital and Shengjing Hospital of China Medical University. The data was collected between 2014 and 2023 using two different ECG machines, the GE MAC800 and the NIHON KOHDEN ECG-2550. Cases include kids who are an average of 2.12 ± 1.50 years old, and CHD cases make up a big part of that group. The information shows real-life medical situations. Most of the CHDs are related to VSD, ASD, PDA, and TOF. As long as the data annotations meet ICD-10 standards, they are clinically relevant. Multi-centered sources and a variety of devices make the dataset more reliable and useful for real-world use.

### 4.6 Non-clinical dataset

Non-clinical data can enhance CHD recognition through ML by offering additional insights beyond conventional clinical measurements. Socioeconomic status, parental health behaviors, environmental exposures, healthcare access, and demographic data can be incorporated into ML models to improve predictive accuracy. By integrating non-clinical characteristics, ML algorithms can reveal concealed patterns and correlations that may not be evident from clinical data alone, providing a more comprehensive and precise risk assessment for early identification and management. Three such datasets are reported in the litrature which we present in the following pargraphs.

This dataset (58) comprises electronic health records from 567,498 pediatric patients at the Guangzhou Women and Children's Medical Center. The records encompass comprehensive clinical data, including diagnoses classified per the ICD-10, symptoms, history of present illness, physical examination results, and laboratory test outcomes. The dataset encompasses a varied pediatric population with a median age of 2.35 years and includes a broad spectrum of both common and unusual pediatric illnesses. All patient records were anonymized to guarantee adherence to data privacy standards.

This dataset (59) comprises records of 33,831 live births collected between 2006 and 2008 in Shanxi Province, China. Among these, 78 individuals were identified with CHD abnormalities. The dataset exhibits significant imbalance, with the majority of entries classified under the non-CHD category. The research sought to forecast CHD risk by examining maternal health and lifestyle determinants, encompassing characteristics such as maternal age, income, familial history, medical history, nutrition, medication consumption during gestation, and environmental exposures (e.g., X-rays).

## 5 ML/DL algorithms used for CHD

It is not easy to organize all ML methodologies for CHD recognition into a single taxonomy. The difficulty lies in the variety of data formats employed for problem analysis. The methodologies employed to assess ECG signals are distinct from those utilized for the examination of sound modalities. Therefore, we do not adhere to a specific taxonomy to address CHD through ML. Table 4 clearly indicates that, throughout the years, researchers have shown greater interest in DL algorithms rather than classical ML in the development of CHD-ML models. Notably, 60 out of 74 papers (81%) focused on addressing CHD using DL, while only 14 papers (19%) utilized classical ML methods.

**Table 4:** CHD databases reported in state-of-the-art (SOA).

References	ML method	Evaluation results
2025		
Lee et al. (104)	CNN	*S*_*c*_: 0.72, *A*_*c*_: 0.67, AUC: 0.81
Haq et al. (105)	CNN	*P*_*c*_: 0.98, *A*_*c*_: 0.98, *F*_1_:0.98, *S*_*c*_: 0.99
Mayourian et al. (106)	CNN	*R*_*c*_: 0.79, *AUC*: 0.17
Shi et al. (107)	TML	*PPV*: 0.95, *FN*: 33
Sharifi et al. (108)	EfficientNetV2	*A*_*c*_: 0.91, *P*_*c*_: 0.90, *S*_*c*_: 0.93
2024		
Jia et al. (37)	RF, KNN	*F*_*c*_: 0.90 and 0.934
Qiao et al. (86)	Hybrid	*A*_*c*_: 0.96, *P*_*c*_: 0.89, *R*_*c*_: 0.99
Cheng et al. (74)	CNN	RUC: 0.915, *S*_*c*_: 0.917, *S*_*p*_: 0.90
Zhixin et al. (42)	3D/2D U-Net	*S*_*c*_: 0.82, *S*_*p*_: 0.87, *A*_*c*_: 0.86
Han et al. (43)	ResNet18	*A*_*c*_: 0.80, ROC: 0.85
Xuu et al. (109)	ResNets	AUC: 0.94, *S*_*c*_: 0.97, *S*_*p*_: 0.98
Cheng et al. (100)	ResNet18	AUC: 0.99, *A*_*c*_: 0.99
Marelli et al. (110)	GBDT, SVM	*S*_*c*_: 0.99, *S*_*p*_: 0.97, *F*_*c*_: 0.99
Pace et al. (51)	3D U-Net	*DS*_*c*_: 0.87
Siefkes et al. (60)	RF, LR, GB	*A*_*c*_: 0.92
2023		
Xuu et al. (109)	CDLM	*S*_*c*_: 0.91, *S*_*p*_: 0.92, PPV: 0.90
Pachiyannan et al. (86)	ANN	*A*_*c*_: 0.71, *S*_*c*_: 0.63, *S*_*p*_: 0.77
Sapitri et al. (111)	YOLOv7	*P*_*c*_: 0.82
Arnaout et al. (112)	ResNet50	AUC: 0.92, *A*_*c*_: 0.91
Yang et al. (33)	ResNet50, LSTM	*A*_*c*_: 0.94, *S*_*c*_: 0.93, *S*_*p*_: 0.95
Jia et al. (37)	CNNs	AUC: 0.99, *S*_*c*_: 0.95, *S*_*p*_: 0.96
Jiang et al. (113)	CNNs	AUC: 0.99, *S*_*c*_: 0.95, *S*_*p*_: 0.96
**References**	**ML Method**	**Results**
Rima et al. (112)	RNN	*A*_*c*_: 0.97
Yang et al. (33)	Hybrid	*S*_*c*_: 0.82, *S*_*p*_: 0.87, *A*_*c*_: 0.86
Tan et al. (45)	Bayesian models	*P*_*c*_: 0.94, *R*_*c*_: 0.95
Xu et al. (109)	BiConvLSTM	ROC: 0.79, *A*_*c*_: 0.69
2022		
Qio	RLDS (CNNs)	*P*_*c*_: 0.93, *R*_*c*_: 0.93, *F*_*c*_: 0.93
Gearhart et al. (114)	CNNs	*A*_*c*_: 0.90, PPV: 0.98, *S*_*c*_: 0.94
Truong et al. (61)	RF	*S*_*c*_: 0.85, *S*_*p*_: 0.88, PPV: 0.55
Goretti et al. (115)	CNNs	ROC: 0.79
Liu et al. (32)	Residual CNNs	*S*_*c*_: 0.93, *S*_*p*_: 0.99, *P*_*c*_: 0.88
Xu et al. (31)	RF, AdaBoost	*A*_*c*_: 0.95, *S*_*c*_: 0.94, *S*_*p*_: 0.96
Oliveira et al. (116)	CNNs	*S*_*c*_: 0.94, *S*_*p*_: 0.95, *F*_*c*_: 0.94
Nurmaini et al. (101)	DenseNet201	*S*_*c*_: 0.91, *S*_*p*_: 0.92, *A*_*c*_: 0.92
Kavitha et al. (98)	MLDDP	*A*_*c*_: 0.98
Sakai et al. (66)	Autoencoder	ROC: 0.97
Wu et al. (117)	YOLOv5	*P*_*c*_: 0.96, *R*_*c*_: 0.85
2021		
Komatsu et al. (118)	CNN	ROC: 0.78
Xu et al. (103)	3D-CNNs	*A*_*c*_: 0.81
Wang et al. (119)	CNNs	*A*_*c*_: 0.93 (binary), 0.92 (three-class)
Lv et al. (34)	SVM	*S*_*p*_: 0.97, *S*_*c*_: 0.89, *A*_*c*_: 0.96
Belinha et al. (35)	ANN, DT	AUC: 0.76
He et al. (65)	CNN	*DS*_*c*_: 0.98
2020		
Tan et al. (120)	SVM	*F*_*c*_: (0.72–0.87)
Qiao et al. (121)	YOLOv4	*P*_*c*_: 0.91, *R*_*c*_: 0.97, *F*_*c*_: 0.94
Gong et al. (122)	GANs	*A*_*c*_: 0.85
Karimi et al. (102)	GANs	*DS*_*c*_: 0.91 and 0.84
Thomas et al. (62)	SVM	*A*_*c*_: 0.85
Diller et al. (77)	PG-GAN, U-Net	*DS*_*c*_: 1.0
Rani et al. (64)	ANN	*A*_*c*_: 0.99
2019		
Oliveira et al. (63)	HSMM	*A*_*c*_: 0.92
Bozkurt et al. (72)	CNNs	*S*_*c*_: 0.84, *S*_*p*_: 0.78, *A*_*c*_: 0.81
Lili et al. (123)	NN	*S*_*c*_: 0.88, *S*_*p*_: 0.93
Linag (58)	CNN + LR	*A*_*c*_: 0.90
2018		
Luo et al. (59)	SVM, RF, LR	TPR: 0.69, TNR: 0.94, *A*_*c*_: 0.94
Liu et al. (81)	LSTM, RNN	*A*_*c*_: 0.98
Vullings et al. (73)	CNNs	*A*_*c*_: 0.76

### 5.1 Conventional ML methods

Different TML techniques have been used for CHD recognition. Feature engineering for classical ML algorithms is a more complex and labor-intensive procedure. TML consists of multiple distinct phases, namely pre-processing, feature extraction, and classification.

The research conducted by Xu et al. (50) employs cardiac signals to classify CHD. The authors have identified a variety of diverse and extensive characteristics. The authors employed frequency domain data and wavelets to construct traditional classifiers within an RF framework. The authors of the research contend that SOA datasets have markedly enhanced outcomes. The researchers replicated the anatomical architecture of the hearts and principal blood arteries of 29 youngsters with CHD anomalies. The authors reported better results compared to previous findings.

The work in Jia (37) leverages the ZCHSound database, an open-source pediatric CHD heart sound dataset. Classification was performed using RF and KNN. Evaluation criteria included *A*_*c*_, *S*_*c*_, *S*_*p*_, and *F*_*c*_. The *F*_*c*_ attained for high-quality data was 0.90 for binary classification (normal vs. CHD) and 0.934 for multi-class classification (normal, ASD, VSD, PDA, and PFO). The F1-score for low-quality data was 0.62 for binary classification and 0.46 for multi-class classification. Some more papers that are using SVM, RF, LR, GB, and Adaboost classifiers with certain features can be explored in (31, 32, 60–62).

The manuscript (63) presents an approach for heart sound segmentation utilizing an HSMM with variable sojourn time parameters. The HSMM analyses PCG signals by utilizing aspects including homomorphic and Hilbert envelograms, wavelet-based characteristics, and power spectral density. The datasets used during this work is PhysioNet. The evaluation metrics include *A*_*c*_, *S*_*c*_, *S*_*p*_, and *F*_*c*_. The authors report an F1-score of 92% on the PhysioNet dataset. The method is resilient across datasets, facilitating efficient transfer learning across training and testing sets.

Another work reported in Rani and Masood (64) employs an ANN as the principal ML technique for CHD recognition in prenatal contexts, utilizing non-clinical data from expectant moms. The dataset comprises 33,831 live birth records gathered from Shanxi, China. The ANN attained an exceptional *A*_*c*_ of 99%. The authors improved *A*_*c*_ of their previous own work, (previously using SVM with an *A*_*c*_ of 0.947). Some more excellent papers that are exploring ANNs for CHD recognition can be studied here (34, 35, 65, 66).

### 5.2 Deep learning based methods

This section summarizes various DL models developed for CHD recognition. The progression of DL algorithms has significantly transformed CV methodologies. Table 4 indicates that the predominant algorithms used in contemporary research are based on DL methods. We present a summary of the most commonly used DL strategies for CHD recognition in this section of the paper.

#### 5.2.1 Convolutional neural networks

The architecture of CNNs is modeled inspired from human visual system (67, 68). The CNNs framework has two phases: feature extraction and classification. The feature extraction consists of multiple hierarchical structures within convolutional, activation, and lastly pooling layers. The convolutional layer generates features that are based on input data through the convolution process. The model's complexities are reduced, enabling a more simple training procedure due to the reduced number of hyperparameters requiring adjustment through the spatial sharing of the kernel throughout the entire input data in each convolutional layer. A pooling layer reduced the resolution of the feature map, hence achieving shift invariance. The pooling layer is situated subsequent to each convolutional layer. In the classification step, the feature maps generated during feature extraction are utilized with a SoftMax function and fully connected layers. The authors of Gao et al. (69) shown that the fully linked layer can be eliminated by employing a global average pooling layer. SoftMax is frequently employed for classification tasks (70, 71), while SVM and other classifiers have also been used. Several exemplary research studies that are using CNNs for CHD recognition are discussed below.

- A research paper that leverages time-frequency representations of PCG information and CNNs is reported in Bozkurt et al. (72). The principal feature extraction technique included in the study is sub-band envelopes, an innovative method in this field, which has demonstrated superior performance compared to traditional features like Mel Frequency Cepstral Coefficients and Mel-spectrograms. The analyzed datasets consist of heart murmur signals, which contain pediatric PCG recordings with clinical annotations. Another publicly available dataset, PhysioNet, has also been used. The evaluation measures reveal better performance, with *S*_*c*_ 0.845, *S*_*p*_ 0.785, and *A*_*c*_ 0.815.
- Similarly, Vullings (73) uses deep NNs that include six convolutional layers and residual connections to examine fetal ECG data for CHD recognition. Feature extraction entailed the generation of a three-dimensional vectorcardiogram from fetal ECG signals obtained through maternal abdominal electrodes. The dataset consisted of 386 fetal ECG recordings, encompassing 266 from healthy fetuses and 120 from those with CHD. The proposed method attained an *A*_*c*_ value of 0.76.
- A CNN-based method for CHD is proposed in Chen and Zhang (74). The authors present a method named CHDdECG. The type of data used is ECG signal. Along with DL wavelet transform is also used. The authors subsequently amalgamate these characteristics with substantial human-concept variables. CHDdECG was assessed on a dataset of 65,869 samles obtaining a good AUC value. The proposed method was assessed using two separate external datasets, comprising 7,137 and 8,121 cases.
- Kavitha et al. (37) proposed a method termed multilayer deep detection perception. The authors of the paper employed ultrasonic imagery for their investigation. The model derives features using a multilayer DL architecture with several perceptron layers. The authors assert that the suggested model's performance on the SOA dataset is superior.
- A narrative literature review on the similar topic has been published in 2023 by Ainiwaer et al. (75). The paper discussed the integration of AI with electronic stethoscopes to improve the diagnosis of CHD through the analysis of heart sounds (PCGs). It explains the foundational concepts such as normal heart sounds (S1–S4) and murmurs, and outlines AI steps like preprocessing, segmentation, feature extraction, and classification using either end-to-end deep learning or traditional ML methods. The authors compared different types of stethoscope technologies and highlight studies reporting 78%–99% accuracy in detecting CHD types such as ASD, VSD, and PDA. They highlighted that AI can be a potential cost effective screening tool for CHD detection along with Echo, while acknowledging limitations such as data scarcity and lack of standardization. The Ainiwaer et al.'s review (75) only focuses on audiological methods for VHD and CHD using heart sounds, and does not use a systematic methodology or meta-analysis. This review only covers the literature up to 2023, with particular focus on signal processing techniques and device compatibility. In contrast, the propose SLR utilizes a strict PRISMA-guided methodology, analyzing 432 papers (2018–2025) and extracting metadata from 74 papers. The propose SLR cover a wide range of CHD diagnosis methods, it also includes global trends, funding sources, datasets, algorithms, evaluation metrics, and ethical considerations, establishing itself as a comprehensive and up-to dated resource for ML experts and practitioners in this underexplored domain.

#### 5.2.2 Generative adversarial network models

A GAN is a category of ML frameworks intended to produce novel data samples that mimic a specified dataset. GANs comprise two neural networks: the generator, responsible for producing synthetic data, and the discriminator, tasked with assessing the veracity of the input. The generator seeks to refine its proficiency in producing realistic data, while the discriminator augments its capacity to differentiate between authentic and fake data. Unlike CNNs, which primarily focus on image classification, object identification, and feature extraction, GANs are specifically designed for data production. We believe that GANs will be exceptionally proficient at CHD recognition, particularly in contexts characterized by restricted data availability.

- A paper in Gong (76) proposes the DANomaly and GAN-based model for fetal CHD recognition. Expert cardiologists annotate the end-systolic phases of four-chamber heart video slices to extract features. Evaluation metrics reveal that the method achieves 85% recognition accuracy, outperforming expert cardiologists and SOA networks.
- A work proposed in Diller et al. (77) utilizes GAN to produce synthetic cardiac MRI images for patients with CHD, particularly those diagnosed with TOF. The GAN was trained using 303 MRI datasets from patients, generating over 100,000 synthetic images. The synthetic images were utilized to train U-Net segmentation models for the segmentation of heart chambers. The evaluation measures comprised the Dice coefficient and percentage area variation. U-Nets trained on synthetic data had comparable performance to those trained on authentic patient data, with a segmentation accuracy discrepancy of less than 1%, so illustrating the viability of utilizing synthetic datasets for training purposes.

#### 5.2.3 Recurrent neural networks

RNNs and Long Short-Term Memory (LSTM) are a kind of neural networks (NNs) engineered to capture long-range relationships in sequential data through the use of memory cells (78). LSTMs are predominantly used for time series and NLP applications, but they can facilitate image recognition by examining sequences of image data, including video frames or segmented image characteristics (79). LSTMs improve tasks like action detection in movies and image sequence prediction by maintaining contextual information; hence, they augment conventional spatial analysis techniques in image-centric applications (80).

- The research paper in Liu et al. (81) utilizes LSTM for the classification of CHD based on ECG signals. Symbolic aggregate approximation does feature extraction, which reduces the number of dimensions in the data and turns sequences into symbolic representations while keeping important characteristics and lowering the amount of work that needs to be done on the computer. The dataset comprises 5,000 preprocessed heartbeats annotated with class values from automatic annotations. The model achieves an *A*_*c*_ of 98.4%. Symbolic aggregate approximation preprocessing markedly improves classification performance and efficiency, yielding optimal results.
- An LSTM based framework has been introduced by Ng et al. (82) for CHD classification. The authors of this study have presented a novel and unique feature extraction method. This study integrates both chromatic and textural attributes. Better results are reported by this paper as compare to previous results on their own dat.
- A recent study on CHD recognition utilizing DL is presented in Garcia-Canadilla et al. (83). This paper introduces an ML-based technique for CHD recognition, termed ML-CHDPM. This method focuses on combining LSTM with specific attention mechanisms. This study presents an approach that integrates CNN, bi-directional LSTM, and attention mechanisms. The authors claim that the new model has enhanced previously published results.

#### 5.2.4 Hybrid models

Hybrid models integrate traditional machine learning (TML) methods with DL to capitalize on the advantages of both paradigms. These models frequently employ conventional ML techniques, such as DT or SVM etc., to analyse structured or tabular data, whereas DL models, such as neural networks, manage unstructured data, like images or text (84). In image recognition tasks, a DL model can extract high-level features from images, whereas a conventional ML method may classify these features according to established criteria (85).

- This study (86) employs a hybrid ML method combining CNNs, BiLSTM for the early detection and diagnosis of CHD using ECG signals. The dataset includes ECG records sourced from the PhysioBank repository and other publicly available databases, with preprocessing steps involving Z-score normalization and segmentation into 2-second intervals. The proposed ML-CHDPM model achieves superior evaluation metrics, with an *A*_*c*_ of 0.96, *P*_*c*_ of 0.89, *R*_*c*_ of 0.99, and *S*_*p*_ of 0.90.
- A paper in Tan et al. (45) details the development of an AI-based CHD model (CHDNet). This model works as a binary classifier that analyses echo to identify abnormalities in the heart. The authors claim that CHDNet's effectiveness is equal or better than that of medical specialists. The authors propose two techniques: Bayesian inference and dynamic neural feedback. These methods are employed to precisely evaluate and improve the diagnostic reliability of AI. The preliminary method enables the neural network to generate an evaluation of its reliability rather than a single forecast outcome.

## 6 Results and discussion

### 6.1 Features extraction methods

Current feature extraction techniques present significant challenges for ML specialists in addressing CHD using traditional methods. Preprocessing and feature extraction are critical components in building effective ML systems, and the choice of preprocessing technique depends on the specific characteristics of the dataset. The suitability of a particular technique often varies depending on the type of data being analyzed, as observed across various studies. These methods exhibit considerable variability, and the lack of standardized reporting frameworks adds to the complexity. This variability is partly due to the heterogeneity of CHD-related data; for instance, approaches effective for image data may not be appropriate for audio or tabular data.

### 6.2 Systems limitations

The quality of training data plays a pivotal role in determining the efficacy of systems used for CHD recognition. Both the training data and the extracted features significantly impact system performance, with superior training data leading to better outcomes. However, most current systems must meet specific criteria to achieve accurate functionality. Failure to adhere to these criteria can result in errors, such as inaccurate disease identification. For instance, overfitting remains a common issue, particularly in DL and traditional ML methods. To address these challenges, researchers should focus on developing adaptive systems that offer greater flexibility in their requirements.

### 6.3 Previous results comparison

An analysis of Table 4 highlights the disparity in performance between TML techniques and contemporary DL methodologies. Data from Table 4 shows that DL techniques consistently outperform TML methods, especially when applied to complex datasets. Interestingly, in some cases, influence-based systems exhibit better performance compared to DL methods. This underscores the importance of developing a more comprehensive understanding of DL algorithms and their applications. Studies such as Chen and Zhang (74) and Pachiyannan et al. (86) show that DL approaches deliver significantly improved results for complex and extensive databases, further reinforcing their potential. However, Table 4 also reveals varied outcomes for CHD recognition when comparing the effectiveness of traditional ML approaches.

This survey presents a detailed (Table 4) summarizing data from prior studies utilizing multiple performance criteria, including accuracy, sensitivity, specificity, and others. Nonetheless, we refrained from directly comparing the outcomes of the experimental section due to two reasons. First, the majority of studies utilized disparate datasets for their experiments, complicating the establishment of a meaningful comparison. There is very less repetition of the same database by other authors. Secondly, the absence of comprehensive information concerning dataset configurations, including the particular participants utilized for training and testing, exacerbates the challenge of direct evaluation.

We conducted subgroup analyses by organizing studies from our SLR according to shared modalities, tasks, and metrics, utilizing data from 74 reviewed papers where sufficient information was available. Our analysis focuses on:

- PCG for binary classification of CHD vs. normal,
- ECG for binary classification, and
- Echo for segmentation and measurement tasks.

The typical performance ranges were determined from pooled or representative values across different studies, such as those using public datasets like PhysioNet or CirCor for PCG and ECG, and custom fetal or pediatric cohorts for Echo. A summary of the analysis is presented in Table 5.

**Table 5:** Performance ranges of ML-models for modalities PCG, ECG, and echo.

Modality	Task	# Studies	Main models	Key performance metrics
PCG	Binary task	6	CNN, SVM	Sensitivity: 85%–99% (~91%) Specificity: 86%–99% (~93%) AUC: 0.82–0.99 (~0.95) Accuracy: 87%–98%
ECG	Binary task	5	Hybrid CNN-LSTM	Sen.: 83%–99% (~90%) Spec.: 81%–95% (~88%) AUC: 0.76–0.96 (~0.89) Accuracy: 76%–96%
Echo	Segmentation	8	U-Net, CNN	Dice 0.83–0.92 Sens. 76%–97%, Spec. 76%–99% MAE ~4–5, AUC 0.91%–0.99%

Measurement tasks, such as left ventricular ejection fraction (LVEF), yield mean absolute error (MAE) of approximately 4%–5%. These results have been incorporated into a new subsection in Section 6, along with Table X, for improved clarity.

### 6.4 Dataset heterogeneity, generalizability, and bias concerns

A major concern in CHD-ML is dataset heterogeneity and limited generalizability. The dominance of single-center, China-based cohorts ( 46.3% of studies) and population-specific data raises risks of ethnic, geographic, and socioeconomic biases, as well as device-specific artifacts (e.g., varying ECG/ultrasound hardware). Models trained on such data may not generalize reliably to other ethnicities, healthcare systems, or resource-limited settings, potentially widening disparities if deployed without rigorous multi-center/external validation across diverse populations. Future work must prioritize multicenter, multi-ethnic, and multi-device datasets (including underrepresented regions) and explicit bias assessments to ensure equitable and robust CHD-ML systems.

### 6.5 Data augmentation and synthetic data

We anticipate a shift in the recognition of CHD toward innovative DL algorithms. DL methods have training challenges due to insufficient ground truth data. Efficient information transmission could provide a feasible resolution to this issue (87). It is prudent to explore alternatives such as self-directed learning and supervised learning (88). Another potential area for improvement involves the utilization of data augmentation (89) and the implementation of foveated architectural strategies (90). In DL architectures, data augmentation mitigates the issue of inadequate data.

### 6.6 Vision transfer model—to be explored for CHD-ML

DL methodologies have attained exceptional outcomes in diverse imaging assessments. Nonetheless, the challenges of substantial parameter size and diminished throughput restrict their clinical uses. A ViT model is the next thing to be explored with CHD recognition. ViT models can provide numerous benefits for CHD recognition in contrast to conventional ML and DL techniques. ViTs use a self-attention mechanism to find global dependencies in data, which makes them better at finding complex patterns and subtle anomalies that are common in CHD datasets. CNNs, on the other hand, rely on local receptive fields for feature extraction. Moreover, ViTs lack the inductive biases characteristic of CNNs, enabling them to adapt flexibly to many modalities, including echo, X-rays, MRIs, etc. This is essential for CHD recognition, because anatomical variances may be substantial. ViTs scale effectively with large datasets when pre-trained on huge collections and subsequently fine-tuned on specialized medical datasets, facilitating strong feature representation despite insufficient label data. These models can also combine different types of data (for example, imaging data with clinical metadata) to make a more complete diagnosis, which makes the performance of CHD recognition models better. These characteristics render ViT a promising instrument for enhancing CHD detection and diagnosis in clinical environments.

### 6.7 Gaps in research

A major gap exists in the use of AI for the diagnosis and treatment of patients with CHDs across their lifespan. The utilization of AI in CHD has been restricted by the lack of CHD-specific datasets with labels for model training, the complicated modeling demands caused by diverse clinical characteristics and age-related pathophysiological changes, and the fragmented nature of data within center-specific repositories. Furthermore, at baseline, information regarding particular rare forms of CHDs is limited, requiring multidisciplinary collaboration to compile sufficient datasets. Substantial deficiencies in clinical training, knowledge, experience, and familiarity with AI exist.

CHD continues to be an inadequately explored area for both cardiovascular and ML experts (46, 91). The region remains unexplored due to various intricate factors. Hospitals generate significant quantities of data, encompassing clinical information and data from electronic health records. The continuous progression of big data is crucial in healthcare management as it enables the analysis of large datasets to improve illness treatment, determine appropriate therapeutic dosages, and generate predictions (92). Healthcare produces significant data; nevertheless, a considerable portion remains underused due to challenges in storing, organizing, and understanding complex datasets that often involve multidimensional and nonlinear relationships among variables. The application of these datasets, particularly in uncommon diseases such as CHD, in conjunction with AI predictive models, can assist in identifying individuals at risk of having children with CHD (92–95).

## 7 Conclusion

The integration of AI into CHD research and treatment has brought transformative advancements, demonstrating its potential to revolutionize diagnosis, prognosis, and therapeutic interventions. This comprehensive survey has provided an in-depth review of the AI methodologies applied in CHD recognition, emphasizing the significant progress made and their profound impact on clinical practices.

By analyzing state of the art techniques, ranging from TML methods with manually engineered features to advanced DL frameworks, we have outlined the evolution of AI approaches in CHD management. This study also highlights critical challenges, such as the need for diverse and complex datasets to enhance the performance and generalizability of DL models. Furthermore, the curated compilation of publicly available resources, algorithms, and methodologies serves as a valuable reference for fostering future research and innovation in this domain.

The success of AI in CHD care relies heavily on interdisciplinary collaboration. Partnerships among AI researchers, clinicians, bioinformaticians, and industry professionals are essential for translating technological advancements into clinically viable tools. Such collaborations can ensure that AI-driven solutions are tailored to real-world challenges, focusing on patient-centered outcomes and seamless integration into healthcare systems.

AI's potential for personalized CHD therapy is particularly promising. By leveraging patient-specific data, AI can enable precision treatment plans, optimize therapeutic efficacy, and improve overall patient outcomes. Predictive models driven by AI can minimize trial-and-error approaches, allowing clinicians to deliver more effective and targeted interventions.

While the progress is undeniable, the path to fully realizing AI's potential in CHD care remains a work in progress. Addressing existing challenges, fostering continuous innovation, and strengthening collaborations are imperative for overcoming current limitations. With sustained efforts, AI has the capacity to redefine CHD management, significantly improving the quality of life for patients and offering hope for a healthier future.

## Data Availability

Publicly available datasets were analyzed in this study. Not all the datasets are publicly available. Some of these datasets are publically available.
