# Supplementary material for: Congenital heart disease diagnosis using machine learning: a systematic literature review
Source: Front Med (Lausanne). 2026 Apr 21;13:1757468. doi: 10.3389/fmed.2026.1757468 (PMC13139176; doi:10.3389/fmed.2026.1757468)
Supplement: Supplementary file 1 [file Data_Sheet_1.pdf]

Supplementary Appendix S1
Search Strategy and Selection Details

This appendix provides a reproducible description of the search strategy, databases, search strings, dates, de-duplication process, and record flow for the systematic literature review titled "Congenital Heart Disease Diagnosis using Machine Learning: A Systematic Literature Review". The methods adhere to PRISMA 2020 guidelines and the PRISMA-S extension for reporting literature searches.

1 Databases Searched

The following databases were searched to minimize single-source bias and ensure comprehensive coverage of biomedical, clinical, multidisciplinary, and engineering literature:

- Scopus
- PubMed (including MEDLINE)
- Web of Science Core Collection

No other databases (e.g., Google Scholar, IEEE Xplore beyond WoS coverage, Embase) were searched, as the selected trio provides sufficient overlap and high-quality peer-reviewed content for this topic. Google Scholar was excluded due to lack of reproducible advanced syntax, controlled vocabulary support, and challenges in systematic de-duplication.

2 Search Dates

- Initial searches: Conducted between January 15, 2025 and April 10, 2025.
- No update/forward citation search was performed beyond the predefined publication window of 2018–2025.

3 Search Strategy and Strings

The search combined terms related to **congenital heart disease** (including synonyms and specific defects) with terms for **machine learning / artificial intelligence / deep learning**. Filters for publication years (2018–2025), English language, and document types (primarily articles and reviews) were applied where possible.

3.1 Scopus

```
TITLE-ABS-KEY ( ( "congenital heart" OR "congenital cardiac" OR "congenital card
AND PUBYEAR > 2017 AND PUBYEAR < 2025
AND LANGUAGE ( english )
AND ( LIMIT-TO ( DOCTYPE , "ar" ) OR LIMIT-TO ( DOCTYPE , "re" ) )
```

3.2 PubMed

```
( ("Heart Defects, Congenital"[Mesh] OR "congenital heart disease"[Title/Abstrac
AND ("Machine Learning"[Mesh] OR "Artificial Intelligence"[Mesh] OR "Deep Learni
AND ("2018/01/01"[Date - Publication] : "2024/12/31"[Date - Publication])
AND english[Language]
```

3.3 Web of Science Core Collection

```
TS=( ( "congenital heart" OR "congenital cardiac" OR CHD OR "congenital heart di
AND ( "machine learning" OR "deep learning" OR "artificial intelligence" OR "neu
AND PY=(2018-2024)
AND LA=(English)
AND DT=(Article OR Review)
```

Full strings with all synonyms/operators and any minor database-specific adaptations are available upon request from the corresponding author.

4 De-duplication Process

References were imported into EndNote 20 (or equivalent reference manager). De-duplication followed these steps:

1. Import all records from Scopus, PubMed, and Web of Science into a single library.
2. Automated duplicate identification using EndNote "Find Duplicates" (matching on DOI, title + year + authors, journal name; fuzzy matching 90% similarity threshold).
3. Manual review and resolution of remaining potential duplicates (prioritizing records with DOI, complete abstracts, and most recent access date).
4. Final unique records after de-duplication: 4065 (total before screening).

5 PRISMA-Style Flow of Records and Exclusion Reasons

A total of 4065 records were initially identified through database searching. After applying the publication year limit (2018–2025), 2240 records remained, with 1825 studies excluded for being published before 2018 or after 2025. Following restriction to English-language journals, 1920 records were retained, and 320 non-English records were excluded.

Subsequently, subject and journal filtering was performed to remove records from unrelated fields or domains. After this step, 980 records remained, with 940 studies excluded. These excluded studies primarily belonged to areas such as pure Computer Science (without clinical focus), general paediatric diseases,

fetal heart studies without machine learning applications, complex non-congenital heart disease conditions, biotechnologies, biomedical engineering, intelligent health systems, and studies focused solely on mathematics or statistics.

In parallel, non-original research articles were excluded from an intermediate pool of records (n = 1266), resulting in the removal of 654 items, including reviews, conference papers, conference reviews, editorials, notes, and other non-research document types.

Keyword-based filtering was then conducted to retain studies containing core relevant terms. This resulted in 854 records, with 422 studies excluded due to the absence of key terms such as congenital heart disease or defects, AI-assisted auscultation, deep learning, artificial intelligence, echocardiogram, deep neural network, convolutional neural network, fetal screening, pulse oximetry, and ventricular septal defect.

Manual title screening further reduced the number of eligible studies to 432, with 422 records excluded due to irrelevance based on title review. Full-text articles were then retrieved and assessed for eligibility, resulting in 74 studies. Full-text exclusions were not fully quantified in the diagram but included reasons such as lack of direct machine learning application to congenital heart disease diagnosis, inappropriate outcome measures, duplicate content identified during deeper screening, and insufficient methodological quality.

Finally, 74 studies were included in the qualitative synthesis of this systematic review.
